# Validation of the urgency questionnaire in Portuguese: A new instrument to assess overactive bladder syndrome

**DOI:** 10.1590/S1677-5538.IBJU.2017.0147

**Published:** 2018

**Authors:** Rodolfo Pacheco de Moraes, Jonas Lopes da Silva, Adriano Almeida Calado, Geraldo de Aguiar Cavalcanti

**Affiliations:** 1 Divisão de Urologia, Hospital da Polícia Militar de Pernambuco (PMPE), Recife, PE, Brasil; 2 Divisão de Urologia, Hospital Universitário Oswaldo Cruz - Universidade de Pernambuco (HUOC-UPE) Santo Amaro, Recife, PE, Brasil; 3 Divisão de Urologia, Departamento de Cirurgia - Universidade Federal de Pernambuco (UFPE) Cidade Universitária, Recife, PE, Brasil

**Keywords:** Urinary Bladder, Overactive, Validation Studies [Publication Type], Urinary Incontinence, Psychometrics

## Abstract

**Purpose:**

Overactive Bladder (OAB) is a clinical condition characterized by symptoms reported by patients. Therefore, measurement instruments based on reported information are important for understanding its impact and treatment benefits. The aim of this study was to translate, culturally adapt and validate the Urgency Questionnaire (UQ) in Portuguese.

**Materials and Methods:**

Initially, the UQ was translated and culturally adapted to Portuguese. Sixty-three volunteers were enrolled in the study and were interviewed for responding the Portuguese version of the UQ and the validated Portuguese version of the Overactive Bladder Questionnaire short-form (OABq-SF), used as the gold standard measurement for the validation process. Psychometric properties such as criterion validity, stability, and reliability were tested.

**Results:**

Forty-six subjects were included in the symptomatic group (presence of “urgency”), and seventeen were included in the asymptomatic group (control group). There was difference between symptomatic and asymptomatic subjects on all of the subscales (p≤0.001). The UQ subscales correlated with the OABq-SF subscales (p≤0.01), except the subscale “time to control urgency” and the item “impact” from the visual analog scales (VAS). However, these scales correlated with the OABq-SF - Symptom Bother Scale. The UQ subscales demonstrated stability over time (p<0.05), but the subscale “fear of incontinence” and the item “severity” of the VAS did not. All of the UQ subscales showed internal consistencies that were considered to be good or excellent.

**Conclusion:**

The Portuguese version of the UQ proved to be a valid tool for the evaluation of OAB in individuals whose native language is Portuguese.

## INTRODUCTION

The term overactive bladder (OAB) was first proposed by Wein and Abrams to describe “urgency” and “urge-incontinence” symptoms (1). The syndrome is currently defined as the presence of urinary urgency with or without incontinence, usually associated with increased urinary frequency and nocturia, in the absence of an urinary tract infection or other diseases (2). Overactive bladder is a clinical condition with a high prevalence in the general population, with values ranging from 10.8% to 46.9% depending on the population studied and definitions used for the diagnosis (3-5). Community surveys in the United States and Europe indicate that symptoms of OAB are common among both male and female adults, being more prevalent in women than men (30-40% versus 15-30%). OAB in men can be secondary to Bladder Outlet Obstruction (6, 7).

The use of standardized patient-reported outcomes in clinical practice has been shown to improve communication between patients and physicians and may improve the process of treatment selection (8). The usefulness of patient-re-ported outcomes as a measure of treatment effectiveness, which are collected using a previously validated tool, depends on the tool's psychometric properties. Currently, the Overactive Bladder Questionnaire (OAB-q) is the only questionnaire to evaluate the overactive bladder that has been validated to Portuguese (9). The King's Health Questionnaire has also been validated to Portuguese. However, it evaluates mainly the quality of life regarding the presence of urinary incontinence (10, 11). Other questionnaires have been described for overactive bladder, but few value the urinary urgency symptom or are very simple and may not represent the reality of the OAB syndrome (12).

As there are no universally accepted measures for urinary urgency, this new tool could contribute to the evaluation of the impact of OAB, associated or not with urinary incontinence. The “Urgency Questionnaire” (UQ) was proposed by Coyne et al. (13) to assess the context, severity, intensity, and daily life impact of urinary urgency, the cardinal symptom of OAB.

The aim of this study was to translate, culturally adapt and validate the UQ in Portuguese in order to quantify the impact of urinary urgency on the quality of life of patients with OAB.

## MATERIALS AND METHODS

### Translation and cultural adaptation

After receiving authorization from the questionnaire's author, the standard method for survey validation was followed in accordance with adapted international criteria (14) and previously completed validation studies (9, 15, 16).

First, three versions of the UQ were provided by three Portuguese-speaking translators who were fluent in English, and an agreed-upon version in Portuguese was developed by the authors.

Secondly, the translated version was back-translated to English by three translators whose native language was English and were also fluent in Portuguese. The translators were blind to the purpose of this study. An agreed--upon version of the English back-translation was developed and reviewed to ensure that the original content had been maintained and to reveal inconsistencies with the Portuguese version.

Upon the completion of the translation process, a review committee composed by three urologists specialized in voiding disturbances with bilingual professionals created idiomatic and cultural adaptations of the version.

This version was completed after its implementation in a pilot study to determine its cultural appropriateness, and each question was followed with the expression, “I did not understand the question”, as written by Calado et al. (17). After analyzing the data related to the respondent's ability to understand the questionnaire, a final Portuguese version was established.

### Participants

Patients attended in outpatient care at two Urology Centers were invited to participate in the study. Sixty-three patients of both genders with or without lower urinary tract symptoms (LUTS) were enrolled in the study after appointment, and divided into 2 groups: patients who complained of urgency in the last four weeks and patients who denied having urgency. Urgency was defined in the study as “a strong and sudden desire to void and difficulty avoiding going to the bathroom”. All participants who complained of urgency had not been still submitted to OAB syndrome treatment, and they were not treated up to retest.

We excluded patients under the age of 18 or above the age of 80; patients with an active urinary tract infection, pregnancy, neurological diseases, or other medical conditions that could compromise the perception of bladder sensations; and patients with cognitive impairments.

The recommendations of the American Academy of Orthopaedic Surgeons and Institute for Work & Health were used in the cultural adaptation process (18). For the validation study, we used the recommendations of Gorsuch (19) to calculate the sample size required for each domain (minimum subject to item ratio of at least 5:1). Considering the domain “Impact on Daily Activities” that contains the largest number of items (6 items), a minimum sample of 30 patients was required for the process. The institutional ethics committee approved the study, and all of the patients signed an informed consent.

### Questionnaires

The UQ consisted of 15 Likert-type scale items and 4 visual analog scale (VAS) items. The Likert items included 5 responses ranging from “never” to “all the time” and were composed of four subscales (nocturia, fear of incontinence, time to control urgency, and impact on daily activities). The VAS items assessed the severity, intensity, impact, and discomfort of urgency. The higher scores indicated greater intensity of their symptoms. The questionnaire was shown to have validity, reliability and responsiveness (13).

The Overactive Bladder Questionnaire short-form (OABq-SF) has been validated in the Portuguese language, and it was used as the gold standard for this validation study (criterion validity) (9). It consists of 19 questions, which composed two subscales: the “Symptom Bother Scale” (SBS), with 6 questions, and the “Health Related Quality of Life Scale” (HRQL), with 13 questions. A higher score on the SBS indicated a greater degree of dissatisfaction with the condition, and a higher score on the HRQL indicated better quality of life for the patient (20).

### Data collection and statistical analysis

Despite being self-reports, questionnaires were applied rather than interviews to standardize their application and include patients with low levels of education. Sociodemographic and clinical data were collected, and the Portuguese version of the UQ and the OABq-SF were applied. Some patients completed the UQ again 15 days after the baseline.

For the statistical analysis, SPSS software 13.0 and GraphPad Instat® for Windows® and Excel® 2007 were used. A Mann-Whitney U test and paired t-test were used to compare continuous variables, and a Fisher's exact test was used to compare categorical variables. Spearman's rho was used to test the correlation between two continuous variables. The internal consistency of each subscale was tested using the Cronbach's α coefficient. Scores ≥0.7 were considered to be appropriate and excellent when the coefficient was equal to or above 0.8 (21). For all of the tests, an α error <0.05 was considered to be statistically significant.

## RESULTS

### Participants

A total of 63 patients were enrolled in the study; 46 of these were symptomatic, and 17 were asymptomatic (control group). The symptomatic patients had a higher mean age compared to the asymptomatic patients, and there was a higher prevalence of constipation in symptomatic patients than asymptomatic patients (P<0.0001). All of the control subjects were men. There was no difference between the groups for the variables of body mass index (BMI), diabetes, hypertension and history of pelvic/perineal surgery (Table-1).

**Table 1:** Clinical and sociodemographic data of the patients enrolled in the study.

Variables	Symptomatic (n=46)	Asymptomatic (n=17)	P Value
Age (years)	57.72±11.31	51.12±10.75	**0.02** *
Gender (% male)	23 (50%)	17 (100%)	**< 0.01** **
BMI	28.29±7.12	27.96±2.62	0.90*
Diabetes (% yes)	8 (13%)	3 (5%)	1.00**
Hypertension (% yes)	27 (43%)	8 (13%)	0.57**
Constipation (% yes)	19 (30%)	0 (0%)	**< 0.01** **
Perineal/Pelvic Surgery (% yes)	18 (29%)	2 (3%)	0.06

(*) Mann-Whitney test;

(**) Fisher's exact test

### Symptomatic versus asymptomatic participants

The symptomatic patients had higher scores on all UQ subscales and VAS items compared to asymptomatic patients (Table-2).

**Table 2:** Comparative studies of the UQ subscales and VAS items between symptomatic and asymptomatic patients.

Urgency Questionnaire	Symptomatic	Asymptomatic	P value
Subscales	Median (Q1; Q3)	Median (Q1; Q3)	
Impact on Daily Activities	35.42 (19.79; 66.67)	0.00 (0.00; 4.17)	**< 0.001**
Time to Control Urgency	68.75 (42.19; 93.75)	25.00 (0.00; 50.00)	**0.001**
Nocturia	62.50 (37.50; 100.00)	12.50 (0.00; 18.75)	**< 0.001**
Fear of Incontinence	45.83 (16.67; 66.67)	0.00 (0.00; 0.00)	**< 0.001**

**VAS items**			
Impact	7.00 (4.75; 9.25)	0.00 (0.00; 4.50)	**< 0.001**
Severity	7.00 (4.00; 9.00)	0.00 (0.00; 3.50)	**< 0.001**
Intensity	9.00 (6.00; 10.00)	0.00 (0.00; 3.00)	**< 0.001**
Discomfort	9.00 (5.00; 10.00)	0.00 (0.00; 1.50)	**< 0.001**

Mann-Whitney test

### Criterion Validity

The UQ subscales correlated with different scales of the OABq-SF, except for the subscales “time to control urgency” and the item “impact” from the VAS, which did not show a significant correlation with the OABq-SF - HRQL subscale (Table-3).

**Table 3:** Correlation between the scores of the UQ subscales and VAS items and the OABq-SF (criterion validity).

Urgency Questionnaire	Spearman's Coefficient	Spearman's Coefficient	P value
Subscales	(OAB-q SF – Symptom Bother Scale)	(OAB-q SF - HRQL)	
Impact on Daily Activities	0.58		**<0.0001**
		-0.74	**<0.0001**
Time to Control Urgency	0.33		**0.02**
		-0.28	0.06
Nocturia	0.77		**<0.0001**
		-0.65	**<0.0001**
Fear of Incontinence	0.55		**<0.0001**
		-0.56	**<0.0001**
**VAS items**			
Impact	0.32		**0.03**
		-0.25	0.09
Severity	0.62		**<0.0001**
		-0.50	**0.0004**
Intensity	0.53		**0.0002**
		-0.37	**0.01**
Discomfort	0.64		**<0.0001**
	-0.57		**<0.0001**

### Stability

Fifteen patients completed the test-retest. The re-application of the questionnaire occurred 15 days after visit 1. Strong correlations were observed between the Visit 1 and 2 scores on the UQ subscales and VAS items, except for “fear of incontinence” and “severity”. Spearman's correlations ranging from 0.38 to 0.83 on the UQ subscales and from 0.36 to 0.76 on VAS items were found (Table-4). Nevertheless, there were no statistically significant changes from baseline to visit 2 for any of the subscales and VAS items (Table-5).

**Table 4:** Correlation between the scores of the UQ subscales and VAS items at visit 1 and visit 2 (test–retest).

Urgency Questionnaire	Spearman's Coefficient	P value
Subscales		
Impact on Daily Activities	0.82	**0.0002**
Time to Control Urgency	0.70	**0.003**
Nocturia	0.83	**0.0001**
Fear of Incontinence	0.38	0.16
**VAS items**		
Impact	0.52	**0.046**
Severity	0.36	0.18
Intensity	0.66	**0.007**
Discomfort	0.76	**0.001**

**Table 5:** Comparing the scores of the UQ subscales and VAS items at visit 1 and visit 2.

Urgency Questionnaire	Visit 1 Mean (SD)	Visit 2 Mean (SD)	Mean of Difference	t value	p value
Subscales					
Impact on Daily Activities	27.8 (23.0)	30.6 (24.6)	2.8	0.68	0.50
Time to Control Urgency	57.9 (32.1)	58.3 (32.8)	0.40	0.06	0.95
Nocturia	59.2 (34.5)	56.7 (31.6)	-2.5	0.51	0.62
Fear of Incontinence	29.4 (28.5)	30.0 (24.2)	0.6	0.09	0.93
**VAS items**					
Impact	6.2 (2.8)	5.6 (2.7)	-0.6	0.95	0.36
Severity	5.5 (3.1)	5.9 (2.6)	0.4	0.46	0.65
Intensity	6.5 (2.7)	5.1 (2.8)	-1.4	2.14	0.06
Discomfort	6.5 (3.0)	6.2 (2.8)	-0.30	0.54	0.60

Analyses used paired t-tests comparing responses at visit 1 and visit 2.

### Internal consistency

All four UQ subscales demonstrated adequate to strong internal consistency with alphas ranging from 0.73 to 0.85 (Table-6).

**Table 6:** Internal consistency of the Urgency Questionnaire subscales.

Urgency Questionnaire	Cronbach's α	
Subscales	Symptomatic	Symptomatic + Asymptomatic
Impact on Daily Activities	0.842	0.891
Time to Control Urgency	0.852	0.899
Nocturia	0.840	0.879
Fear of Incontinence	0.731	0.817
All items	0.866	0.927

## DISCUSSION

OAB is a highly prevalent condition with a high economic, social and quality-of-life impact on patients. Therefore, instruments are necessary to evaluate it effectively (3-5, 22, 23). Because this common medical condition lacks physiological markers and is defined by symptoms rather than objective measures, it is important to assess treatment outcomes from a patient's perspective (24). Consequently, several patient-reported outcome measures have been developed to assess OAB symptoms and their impact on quality of life. However, in order to reliably assess treatment effectiveness, the instrument used to collect patient-reported outcomes must also demonstrate adequate psychometric properties.

Although the comparative analysis between OAB-q and UQ has been performed in the present study for the validation process, clinical studies have used both questionnaires simultaneously because they may represent distinct tools that identify different aspects of OAB syndrome (25).

The symptomatic patients were older than the control group, which was justified by the increased prevalence of lower urinary tract symptoms with aging (3). The presence of constipation was higher among symptomatic patients than among asymptomatic patients as well. The higher frequency of this condition in females and older patients might justify this result (26, 27).

The UQ is a questionnaire that is validated in English but has not been validated in other languages (13). Another specific-condition questionnaire validated in the Portuguese language was used as the gold standard for establishing the validation criteria. This process evaluated only symptomatic participants, therefore, there was no interference from the absence of asymptomatic women in the present study. Although not all the UQ subscales and VAS items correlated to the scales of the OABq-SF, it does not invalidate the instrument, and it could indicate some differences in what they propose to measure.

Test-retest reliability is the extent to which an instrument produces stable scores over time. Instruments must have adequate test-retest reliability to ensure that they are consistently measuring the construct of interest without excessive measurement error (24, 28). The UQ proved to be stable based on the scores obtained at baseline and after 15 days. Only the subscale “fear of incontinence” and the item “severity” on the VAS showed no correlation between the results from visits 1 and 2. However, there were no statistical changes in any of the subscales from baseline to visit 2. It is also known that how bothersome OAB symptoms are fluctuates, and changes with daily circumstances. This daily fluctuation creates some variation in the measurement of symptom bother and quality of life, which may be considered measurement error (29).

Reliability refers to the consistency with which an instrument measures a construct, and it is considered to be a necessary measurement property of patient-reported outcome instruments (24, 28, 30). The UQ was able to differentiate OAB patients from average, healthy individuals. Internal consistency is an index of consistency across multiple items within a given domain. An adequate consistency was confirmed in the subscales where Cronbach's alpha was calculated. Therefore, the UQ proved to be easily understood when applied as an interview.

The study had some weaknesses. The control group consisted of asymptomatic men only, which could have caused bias. This was due to the greater presence of men without voiding symptoms than women on routine visits. Despite the relatively small sample size, taking into account the significant differences in all of the subscales, conclusions would not have been able to be reached if women had been included in the group. The sample size may have been small to clarify some comparative studies conducted within the limits of the significance level. The calculations, however, were based on previous recommendations for validation studies, which recommend at least 5 patients per item in each domain (19). The questionnaire has been described to be self-reported and should not be administered via interview, even by a previously trained interviewer. Individuals with low educational levels were included, and the method was standardized for illiterate populations. This method has been used in similar validation studies without a significant alteration of the results (16).

## CONCLUSION

The Portuguese version of the UQ represents a comprehensive, valid and reliable instrument for evaluating subjects with OAB, which could be used in clinical studies. Further studies should be conducted to demonstrate its responsiveness.

## APPENDIX Validated version of the Urgency Questionnaire in Portuguese

### Questionário de Urgência Miccional

Esse questionário quer saber sobre as suas experiências quando você sente urgência em urinar. Para cada frase, marque com que frequência você vive esta situação da seguinte maneira: “nenhuma vez, poucas vezes, algumas vezes, na maioria das vezes, ou todas as vezes”. Favor esteja certo(a) de responder cada pergunta marcando um “X” no quadrado apropriado.

### Favor pense na *semana anterior a esta* ao responder à série de afirmações abaixo.

[Table t7]

**Table t7:**

**Durante a semana passada, quando eu senti urgência em urinar** (vontade súbita e forte de urinar e difícil de evitar a ida ao banheiro)…	Nenhuma vez	Poucas vezes	Algumas vezes	Na maioria das vezes	Todas as vezes
1. Consegui controlar esta urgência em urinar por mais de 30 minutos.	□ 1	□ 2	□ 3	□ 4	□ 5
2. Consegui controlar esta urgência em urinar por mais de 20 minutos.	□ 1	□ 2	□ 3	□ 4	□ 5
3. Consegui controlar esta urgência em urinar por mais de 10 minutos.	□ 1	□ 2	□ 3	□ 4	□ 5
4. Consegui controlar esta urgência em urinar por mais de 5 minutos.	□ 1	□ 2	□ 3	□ 4	□ 5
5. Eu perdi urina sem sentir.	1	□ 2	□ 3	□ 4	□ 5
6. Fiquei preocupado(a) em “molhar a roupa”.	□ 1	□ 2	□ 3	□ 4	□ 5
7. Fui acordado(a) durante o sono para urinar.	□ 1	□ 2	□ 3	□ 4	□ 5
8. Não consegui realizar atividades ao ar livre (exemplo: caminhada).	□ 1	□ 2	□ 3	□ 4	□ 5
9. Eu procurei não me afastar de locais que possuem banheiro perto.	□ 1	□ 2	□ 3	□ 4	□ 5
10. Fiquei muito distraído(a) para terminar o que estava fazendo.	□ 1	□ 2	□ 3	□ 4	□ 5
11. Limitei minhas atividades sociais.	□ 1	□ 2	□ 3	□ 4	□ 5
12. Meu sono foi frequentemente interrompido.	□ 1	□ 2	□ 3	□ 4	□ 5
13. Eu me preocupei com a perda de urina.	□ 1	□ 2	□ 3	□ 4	□ 5
14. Tive dificuldades em completar tarefas domésticas diárias.	□ 1	□ 2	□ 3	□ 4	□ 5
15. Tive dificuldades em completar tarefas diárias no trabalho ou na escola.	□ 1	□ 2	□ 3	□ 4	□ 5

**As perguntas nas páginas seguintes pedem que você classifique seu transtorno com a urgência em urinar em uma escala de ‘0’ a ‘10’. Favor responder colocando um “X” em um número da escala que melhor descreve como você se sente, como no exemplo abaixo:**

**Favor completar as questões de 1-4.** [Fig f1]
